# RECODE: Design and baseline results of a cluster randomized trial on cost-effectiveness of integrated COPD management in primary care

**DOI:** 10.1186/1471-2466-13-17

**Published:** 2013-03-23

**Authors:** Annemarije L Kruis, Melinde RS Boland, Catharina H Schoonvelde, Willem JJ Assendelft, Maureen PMH Rutten-van Mölken, Jacobijn Gussekloo, Apostolos Tsiachristas, Niels H Chavannes

**Affiliations:** 1 Dept of Public Health and Primary Care, Leiden University Medical Centre, P.O. Box 9600, Leiden, RC, 2300, The Netherlands; 2 Institute for Medical Technology Assessment, Erasmus University, PO Box 1738, Rotterdam, DR, 3000, the Netherlands; 3 Dept of Primary and Community Care, Radboud University Nijmegen Medical Center, PO Box 9101, Nijmegen, HB, 6500, the Netherlands

**Keywords:** COPD, Disease management, Primary care, Cost-effectiveness, Integrated care

## Abstract

**Background:**

Favorable effects of formal pulmonary rehabilitation in selected moderate to severe COPD patients are well established. Few data are available on the effects and costs of integrated disease management (IDM) programs on quality of care and health status of COPD patients in primary care, representing a much larger group of COPD patients. Therefore, the RECODE trial assesses the long-term clinical and cost-effectiveness of IDM in primary care.

**Methods/design:**

RECODE is a cluster randomized trial with two years of follow-up, during which 40 clusters of primary care teams (including 1086 COPD patients) are randomized to IDM or usual care. The intervention started with a 2-day multidisciplinary course in which healthcare providers are trained as a team in essential components of effective COPD IDM in primary care. During the course, the team redesigns the care process and defines responsibilities of different caregivers. They are trained in how to use feedback on process and outcome data to guide implement guideline-driven integrated healthcare. Practice-tailored feedback reports are provided at baseline, and at 6 and 12 months. The team learns the details of an ICT program that supports recording of process and outcome measures. Afterwards, the team designs a time-contingent individual practice plan, agreeing on steps to be taken in order to integrate a COPD IDM program into daily practice. After 6 and 12 months, there is a refresher course for all teams simultaneously to enable them to learn from each other’s experience. Health status of patients at 12 months is the primary outcome, measured by the Clinical COPD Questionnaire (CCQ). Secondary outcomes include effects on quality of care, disease-specific and generic health-related quality of life, COPD exacerbations, dyspnea, costs of healthcare utilization, and productivity loss.

**Discussion:**

This article presents the protocol and baseline results of the RECODE trial. This study will allow to evaluate whether IDM implemented in primary care can positively influence quality of life and quality of care in mild to moderate COPD patients, thereby making the benefits of multidisciplinary rehabilitation applicable to a substantial part of the COPD population.

**Trial registration:**

Netherlands Trial Register (NTR): NTR2268

## Background

Chronic obstructive pulmonary disease (COPD) is a smoking-related pulmonary disorder, characterized by largely irreversible airflow obstruction, multisystemic manifestations and frequent co-morbidities [1]. According to current guidelines, stable COPD is managed with a combination of different treatment components (e.g. smoking cessation, physiotherapeutic reactivation, self-management, optimization of medication adherence) [1], involving different healthcare providers. Currently, treatment is mostly guided by the severity of airflow limitation [2]. However, COPD is a complex disease, with great variation in symptoms, functional limitations and co-morbidities as well as in progression towards more severe stages [3]. Therefore, the existence of several clinically relevant phenotypes calls for a more personalized approach [4]. Ideally, optimal care of COPD patients requires an individualized, patient-centered approach that recognizes and treats all aspects of the disease, addresses the systemic effects and co-morbidities, and integrates medical care among healthcare professionals and across healthcare sectors [5]. Since professional treatment, hospital admissions and loss of work contribute to the economic burden of disease worldwide, there is much interest in systematically improving the quality of care, while reducing total costs for patients with COPD and other chronic illness. Integrated Disease Management (IDM) programs have proliferated as a means of improving the quality and efficiency of care [6].

The most frequently applied IDM programs in COPD patients are pulmonary rehabilitation (PR) programs. According to a Cochrane systematic review, the effectiveness of PR on exercise tolerance and quality of life is well established [7]. In international reports and guidelines, it is acknowledged that PR is indicated for all individuals with COPD who have decreased exercise tolerance, exertional dyspnea or fatigue, and/or impairment of activities of daily living [1,8,9]. However, widespread access is restricted, due to limited availability of resources and high costs [10-12]. Furthermore, PR programs usually include only the more severe patients and last only for a limited period of time [13], while initial benefits seem to decline over time [14-18]. After returning home, patients are frequently insufficiently motivated to continue a more physically active and healthy lifestyle. Unfortunately, general practitioners (GPs) are rarely involved in PR programs and, as a consequence, are often unable to support program methods after a rehabilitation phase has formally been concluded [13].

**Figure 1:**
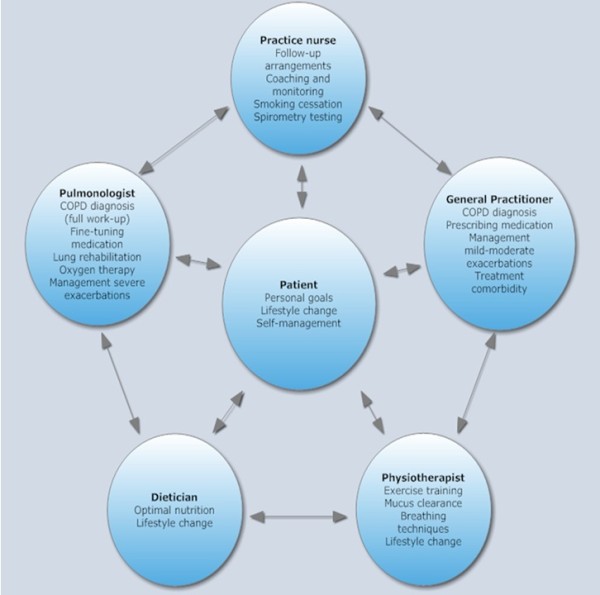
Components of an Integrated Disease Management program for COPD patients in primary care.

We previously argued that when components of PR are integrated into a primary care IDM program, patients can be treated in their home environment. Primary care providers can then be (more) involved as direct coaches of this process [19-21]. To establish such a program of combined interventions, the set-up of a multidisciplinary team is vital, in which different healthcare professionals participate and provide their share in the spectrum of the required care (Figure 1). Ideally, patients and healthcare providers are close partners in IDM, in order to better control daily symptoms and promote self-management. Furthermore, strong cooperation between several disciplines in primary care and mutually agreeable collaboration with secondary and tertiary care are prerequisites for integrated chronic care [19].

Systematic reviews of disease management for COPD patients emphasise the need for well-designed, practical multicenter trials [22,23], including broad representative patient samples [24], with a wide range of physicians and settings to improve external validity [23]. Furthermore, authors of systematic reviews advocate studies designed to evaluate the long-term effectiveness of IDM, [23] and advise more health economic studies across different care settings [24]. When considering the large number of eligible patients for IDM in the community, the potential impact is high. However, no trials have been published that are specifically targeted to measure the cost-effectiveness of IDM in patients recruited in primary care.

Therefore, the aim of the current RECODE (acronym for Randomized Clinical Trial on Effectiveness of integrated COPD management in primary care) cluster randomized clinical trial (NTR 2268) is to assess the cost-effectiveness of an IDM program for COPD patients in primary care in the Netherlands. Based on an earlier controlled clinical trial evaluating the effect of an IDM program in mild to moderate COPD, we found the greatest improvements on quality of life in patients with an MRC dyspnea score >2 [25]. As a result, we based our sample size estimates on the a priori planned subgroup of patients with MRC dyspnea score >2. This article describes the design, rationale and baseline results of this trial.

## Methods/design

### Study objective and design

The RECODE trial is a two-group parallel cluster-randomized clinical trial with a two-year follow-up, conducted in the primary care setting. Our objective is to evaluate the clinical and cost-effectiveness of IDM for COPD patients in primary care. The intervention is delivered by the primary care team, including a GP, practice nurse, physiotherapist and dietician, with a consulting pulmonary physician at hand. To avoid contamination between treatment groups within practices, primary care practices are randomized rather than patients. The Medical Ethics Committee of the Leiden University Medical Centre approved the trial.

## Participants

### GPs

Inclusion of GPs and patients started in September 2010 and was finished in September 2011. Practices were considered as candidates if they were willing to create an integrated COPD management team, in which each member has responsibility for their respective areas of expertise. The practices had to include at least one GP, one practice or extramural respiratory nurse, and one physiotherapist specialized in COPD care. If multiple practices were collaborating (for example with one practice nurse), they formed one cluster which was used for randomization. Our recruitment goal was to enrol representative groups of primary healthcare providers from a broad spectrum of practices in order to enhance external validity. This study was embedded in the Leiden Primary Care Research Network (LEON), which is managed by the department of Public Health and Primary Care of the Leiden University Medical Center. This multi-center research network consists of some 100 general practices in the western region of the Netherlands, in which these practices signed an agreement to collaborate in scientific research.

### Patients

We included all patients who were diagnosed with COPD by their treating physician. We selected patients from electronic medical records (EMRs) of general practices. For all included patients, we attempted to verify the diagnosis by lung function according to the GOLD criteria [1]. If spirometry data were not available, patients were invited to participate for a formal lung function assessment, according to the ATS/ERS guidelines for spirometry [26]. Exclusion criteria consisted of terminally ill patients, dementia or cognitive impairment, inability to fill in Dutch questionnaires, and hard drug or alcohol abusers. We did not exclude patients if a pulmonary physician was considered the main healthcare provider. The GPs checked the selected patients against the formal inclusion and exclusion criteria before the recruitment procedure started. All patients provided written informed consent before participation in the study.

### Intervention

The intervention consists of an IDM program, which is implemented by a multidisciplinary team in general practice. The team consists of at least three members: the GP, the practice nurse, and a cooperating physiotherapist with specific certified training in COPD care. Depending on the team needs, a collaborating pulmonary physician and dietician were added to the intervention team.

We trained the multidisciplinary teams of intervention practices in a two-day course during 2010–2011. During this course, essential components of IDM for effective integrated COPD care in primary care were explained, trained and rehearsed and supervised. Elements of this course are further outlined in Table 1 and included a review of the advice from international guidelines, performing/interpreting spirometry and assessment of disease burden, and motivational interviewing to stimulate a healthier lifestyle including more physical activity and smoking cessation. Furthermore, the healthcare providers were trained in adopting self-management action plans, including early recognition and treatment of exacerbations, encouragement of regular exercise and guideline-based physical reactivation, cooperation and collaboration with secondary care, and instructions in dietician support for nutritionally depleted patients. In addition, they were trained in how to use feedback on process and outcome data to guide and implement guideline-driven integrated healthcare. This continuining medical education course was developed according to recent national and international guidelines [1,27] and was provided by teachers with hands-on experience with the program. At the end of the course, the team designed a time-contingent individual practice plan, agreeing on steps to be taken in order to integrate a COPD IDM program into daily practice. Intervention practices were free in the fulfilment of their individual plans, as long as they were feasible and relevant for the practice. After 6 and 12 months, there was a refresher course for the intervention practices.

**Table 1:** Components of IDM included in the RECODE course for multidisciplinary teams in primary care

**DM interventions**	**Example**
Optimal medication adherence	Tailoring of advices from international guidelines, e.g. frequent exacerbations necessitate inhaled corticosteroids; daily respiratory complaints necessitate long-acting bronchodilators
Proper diagnosis	Performing and interpreting spirometry, assessment of disease burden using MRC and CCQ
Motivational interviewing	Understanding and making use of patients’ personal goal in physical reactivation and lifestyle changes
Smoking cessation counselling	Review of the recent literature, discussion of bottlenecks, applying behavioural techniques and drug therapy for smoking cessation
Applying self-management plans	Teaching self-management techniques, including early recognition and treatment of exacerbations
Guideline based physiotherapeutic reactivation	Using a patients’ personal goal, referral for physiotherapeutic reactivation in patients with MRC score >2.
Dietary interventions	Early recognition and treatment of nutritionally depleted patients

### Web-based disease management application

During the course, the team learned the details of an ICT program that supports recording of process and outcome measures by access to a flexible web-based IDM application, named Zorgdraad (in English ‘Care Ties’). This application combined a patient and a healthcare provider portal. The patient portal provided patients with disease-specific easy written education, and allows personal goals and personal notes. The healthcare portal left space for a protocol for COPD follow-up guidance, quality of life scores, physiotherapy follow-up and examination, smoking cessation, medication records, and facilitates tailored benchmark reports at 6 and 12 months. These reports were generated by the researchers and sent to the practices to support prioritizing the healthcare needs. An experienced instructor provided the practices during the course with all information about Zorgdraad. An account manager supported the practice nurse and GP on individual use of the program in daily practice. It was intended that practice nurses give the COPD patients directions for use on the patient-portal of Zorgdraad.

All practices were in essence free in the usage of Zorgdraad, and in the fulfilment of their plans. Therefore, not all patients received all components of the program, but individual patient-specific care plans are negotiated by the team, in collaboration with the patient. The intensity of the IDM program depended upon the health status and needs of the patient, resulting in some patients receiving all interventions (e.g. smoking cessation, physiotherapy, nutritional support), while stable patients only had regular 6-monthly or 12-monthly follow-up by nurses. Implementation of the intervention was assessed at 24 months (see “Outcomes”).

### Financial coverage of the intervention

We arranged with the local healthcare insurer that all RECODE patients with dyspnea on moderate or worse exertion (indicated by an Medical Research Council (MRC) score of >2) would be totally reimbursed for the intervention, including physiotherapy.

### Usual care group

The control group consists of ‘usual care’ [28], which is based on the 2007 national primary care COPD guidelines [27]. Instead of the multidisciplinary RECODE course, the practice nurse received a course on technical performance of spirometry in primary care only, in order to divert attention from any of the IDM topics mentioned in Table 1. If the results of our study show that the IDM program could substantially improve the health-related quality of life of COPD patients, we will make the entire set of interventions available to the control group after the study has been completed.

## Outcomes

### Time points

We follow patients at baseline, and at 6 and 12 months with a face-to-face interview. Blinded research nurses administer the questionnaires (Table 2) at specific time points. These interviews take place at the general practice or at the patients’ homes, using the web-based application Zorgdraad. At 9, 18 and 24 months we sent questionnaires by post. In addition, retrospectively the researchers extract data from the patients’ EMRs at 24 months over the complete trial period, regarding prescribed medication.

**Table 2:** Overview of measurements per time point in the RECODE study

**Outcomes**	**Baseline**	**6 m**	**9 m**	**12 m**	**18 m**	**24 m**
**Participants**						
Demographic characteristics	X					
Lung function	X					
Co morbidity	X					
CCQ	X	X	X	X	X	X
SGRQ-C	X	X	X	X	X	X
EQ-5D	X	X	X	X	X	X
SF-36	X	X	X	X	X	X
Smoking behavior, guided smoking attempts	X	X	X	X	X	X
IPAQ	X	X	X	X	X	X
SMAS-30	X	X	X	X	X	X
MRC-Dyspnea scale	X	X	X	X	X	X
Exacerbations	X					X
Costs of health care utilization by patients, part A: Health care use Questionnaire, including direct non-medical costs borne by patients/families	X	X	X	X	X	X
Costs of productivity loss: Absence from work Questionnaire	X	X	X	X	X	X
Costs of health care utilization by patients, part B: Data extraction from medical records (health care utilization, medical treatment)						X
PACIC	X	X	X	X	X	X
**Health care providers**						
ACIC	X			X		
Satisfaction, involvement and implementation of the IDM program				X (IG)		
**IDM program information**						
Development costs of the IDM program						X (IG)
Implementation costs of the IDM program						X (IG)
Performance indicators of practices (see Table 4)	X					X

ACIC: Assessment Chronic Illness Care; CCQ: Clinical COPD Questionnaire; EQ-5D: EuroQol-5D; IPAQ: International Physical Activity Questionnaire; MRC: Medical Research Counsil scale; PACIC: Patient Assessment Chronic Illness Care; SF-36: ShortForm-36; SGRQ-C: Saint Georges Respiratory Questionnaire; SMAS-30: Self Management Scale-30.

IG = intervention group only.

Primary endpoint is at 12 months, when we expect to detect the clinically relevant effect of the intervention [20,25]. Total study duration provides 24 months of follow-up, to assess whether benefits can be maintained.

#### Patients

At baseline, we assessed socio-demographic factors (age, gender, socioeconomic status measured through level of education), marital status, lung function and co-morbidity.

### Primary outcome

The primary outcome measure in this study is health status as measured by the Clinical COPD Questionnaire (CCQ) at 12 months. This questionnaire is a disease-specific, 10-item questionnaire that calculates an overall score and three domain scores: symptoms, functional state and emotional state. Patients are required to respond to each item on a 7-point scale with 0 representing the best possible score and 6 representing the worst possible score. This instrument is proven to be sensitive and valid, and easy to administer in primary care. The minimal clinical important difference (MCID) is −0.4 points [29,30].

### Secondary outcomes

Secondary outcome measurements at 6, 9, 12, 18 and 24 months include (the questionnaire for each outcome is provided in brackets):

1. Measures of changes in health-related quality of life (disease-specific as well as generic), measured by :

a. CCQ

b. St. George Respiratory Questionnaire (SGRQ); designed to measure health impairment in patients with asthma and COPD. The first part produces the symptom score and the second part the activity and impact score. A total score can also be calculated. We use a Dutch version of the SGRQ, and consider a −4 unit change as the MCID for within-group comparison [31].

c. The Euro Qol-5D-3L is a generic, preference-based health-related quality of life questionnaire, with many applications in respiratory disease. It consists of 5 dimensions to describe health (mobility, self-care, usual activities, pain/discomfort, and anxiety/depression) each item with three levels of functioning (e.g., no problems, some problems, and extreme problems). We used the value set derived from the Dutch general population that, when applied to the dimensions of the health state, result in a preference-based utility score that typically ranges from states worse than dead (<0) to 1 (full health), anchoring dead at 0. Besides the descriptive system and the off-the-shelf value sets, the EQ-5D includes a visual analog scale (VAS) where an individual rates his own health on a scale from 0 (worse imaginable health) to 100 (best imaginable health) [32,33].

d. Short-Form Health Survey (SF-36) is a 36-item questionnaire that measures two components (physical and mental component). The physical component consists of four domains of health: physical functioning, role limitations due to physical health, bodily pain and general health perceptions. The mental component consists of role limitations due to emotional problems, vitality, social functioning and mental health [34].

2. Measures of change in patients’ lifestyle, illness behavior and knowledge:

a. Smoking behavior, guided smoking attempts;

b. Taking initiatives, investment behavior and level of self-efficacy, as measured by the Self-Management Scale-30 (SMAS-30) [35];

c. Physical activity, as measured by the International Physical Activity Questionnaire (IPAQ) short form. This is an instrument designed primarily for population surveillance of physical activity among adults. The items in this short form are structured to provide separate scores on walking, moderate-intensity and vigorous-intensity activity. The total score is computed by multiplying the duration (in minutes) and frequency (days) of walking, moderate-intensity and vigorous-intensity activities by its energy requirement to yield a score in Metabolic Equivalent Time (MET) minutes.

3. Measures of change in intermediate patient-related outcomes:

a. Dyspnea, measured by the MRC Dyspnoea Scale [36].

b. Exacerbations: moderate (oral prednisone and/or antibiotic courses), severe (hospitalizations). These data were retrospectively extracted from EMRs at 24 months, over the entire follow-up period.

4. Measures of change in healthcare utilization and costs:

a. Development and implementation costs of the program: time and material resources associated with the training of the healthcare providers and the ICT support (measured at 24 months).

b. Costs of healthcare utilization by patients: including all COPD and non-COPD related cost of a) hospitalization, b) medication, c) caregiver contact, and d) revalidation.

Retrospectively we extract data from EMRs at 24 months over the complete trial period, regarding prescribed medication.

c. Direct non-medical costs borne by patients/families, e.g. travel costs. Costs of productivity loss due to absenteeism/presenteeism at work. This was measured at baseline, and at 6, 9, 12, 18 and 24 months.

5. Measures of change in care delivery process: level of care integration according to patients, measured by the Patient Assessment Chronic Illness Care (PACIC) [37]. This questionnaire was self-reported by patients in both groups and was administered at baseline, and at 6, 9, 12, 18 and 24 months.

#### Healthcare providers

The Assessment Chronic Illness Care (ACIC) questionnaire, which is a tool to measure the level of care integration according to healthcare providers [38], was sent to primary care providers at baseline and is evaluated at 12 months. Furthermore, we use a self-designed questionnaire at 12 months (“Satisfaction, involvement and implementation of the IDM program”) for the primary care team, to measure the level of involvement and implementation of the practice teams with the RECODE intervention at 12 months. This questionnaire comprises questions on the number and type of healthcare providers which were involved in the program, the types of team meetings and local appointments, and the usage of tailored benchmark reports. Furthermore, we requested the number of patients involved in the intervention, and the numbers of components implemented in daily practice. Overall, the healthcare providers are asked to rate the intervention on a 5-point scale, and we ask for details on possible bottlenecks and problems regarding implementation.

#### Current level of care of the practices at baseline

The current level of COPD care was assessed at baseline in all general practices to be able to report any difference in quality of care at 12-months follow-up. Therefore, from the EMRs we extracted the following performance indicators: registration of smoking status and stop-smoking advice, registration of body mass index, assessment of spirometry and inhalation technique in the last year, the number of patients with monitored functioning by means of the CCQ, MRC, or the number of patients with controlled physical activity in the last year.

### Sample size calculation

The primary outcome is the difference in change in the CCQ score between baseline and 12 months between both groups. We used methods for standard sample size estimates for trials that randomised at the level of the individual [39] adjusting for clustering by inflating sample size estimates by the design effect given by 1 + (n-1)ρ, where n is the average cluster size, and ρ is the estimated intraclass correlation coefficient (ICC) [40]. Sample size estimates are based on the mean difference in CCQ between intervention and control group. Using the minimal clinically important mean difference for the CCQ [29], and the upper value of 0.05 from a range of ICC values identified in studies involving the older person in primary care [41], power calculations indicate that 40 clusters of practices with an average of 27 participants per cluster are required. To allow for subgroup analysis in MRC scores 1–2 versus 3–5, in total 1080 participants are need to be randomized to achieve a power of at least 80% with alpha levels of 0.05, including a participant loss to follow-up of 10% or a loss of 4 clusters at 12 months.

### Randomization

Cluster randomization was at the level of the primary care team. The first author recruited the practices, and the selected participants were checked by the GP against formal inclusion and exclusion criteria before the intervention started. To enhance comparability between the intervention and control group, the clusters were matched and randomized by a researcher who was blinded to the identity of the practices. Matching was into pairs according to the following criteria: (i) percentage of patients from ethnic minorities, (ii) type of practice, (iii) practice location (urban/rural), (iv) age of GP, and (v) gender of the GP. Subsequently, the matched practices were randomized to the intervention group or the control group by using a computer-generated random number list.

### Informed consent

Informed consent was provided by the GPs and the patients. The informed consent was acquired before the course took place and the practices started with their intervention.

### Blinding

Because of the nature of the intervention, it is not possible to blind patients and primary care providers to practice group allocation. Therefore, blinded research nurses assess the outcomes. Patients are instructed not to report on their type of management to the outcome assessors.

## Data analysis at baseline

### Non-participation analysis at baseline

We recruited potential participants with an invitation letter including a postal CCQ questionnaire. Returned questionnaires were analysed to investigate if there were differences between participants and patients who fulfilled inclusion criteria, but refused to participate in the trial (non-participants). We compared differences on CCQ scores, sex and age using independent t-tests and chi-square tests.

## Analysis plan

### Analysis of effectiveness at 12 and 24 months

The final analysis of the trial will be carried out on an intention-to-treat basis. The freedom of the clusters to fill in the precise implementation of the intervention will probably relate to the (cost)-effectiveness of the intervention and, therefore, the clustering of patients in GP practices should be taken into consideration in the analysis [42]. Therefore, the results will be investigated with respect to the differences in intensity between and within clusters over time using multi-level analysis.

### Pre-planned subgroup analyses

We will study the influence of age, sex, disease burden (MRC score 1–2 vs. 3–5), disease severity (GOLD stage), and socioeconomic status. The trial was specifically powered on the MRC 1–2 vs. 3–5 subgroup analyses; see ‘Sample size calculation’.

### Economic evaluation at 12 and 24 months

The economic evaluation will be performed according to the internationally agreed guidelines [43] and the national guidelines for pharmacy-economic research [44]. We will calculate the costs from a healthcare perspective and a broad societal perspective, in order to facilitate decision making. The healthcare perspective will include all costs covered by the healthcare sectors budget: development, implementation and healthcare utilization costs. The costs from societal perspective will include travel and productivity costs in addition to the costs from the healthcare perspective to capture (almost) all costs related to the intervention, irrespective of who actually bears them.

The healthcare utilization costs (excluding medication costs), travel costs and productivity costs of patients will be calculated using questionnaires at different time points (Table 2). These questionnaires will collect self-reported cost-related data by patients using a recall period of three months. Additionally, the type and amount of medication from the individual patients will be collected from the GP information systems. The unit costs per medication prescription will be based on the GIP Databank [44]. Time and material resources associated with the training of the healthcare providers, the multidisciplinary team meetings in the GP practices, and the ICT support will be estimated based on course attendance, computer-documented minutes of ICT use, treatment plans, and professional self-report. Finally, the productivity costs will be estimated using the friction method, which implies that the costs of absenteeism will occur only for a fixed (friction) period ending at the moment that the employee is replaced [45].

### Cost-effectiveness (CEA) and cost-utility analyses (CUA)

The relation between the costs and the estimated health outcomes is expressed in cost-effectiveness ratios: (1) costs per QALY, (2) costs per exacerbation prevented, (3) costs per patient with a clinically relevant improvement of at least 0.4 units on the CCQ, (4) costs per patient with a clinically relevant (4 units) improvement on the SGRQ, and (5) costs per patient with a 1 point improvement on the MRC dyspnea scale. Adopting such a wide range of outcome measures in the economic evaluation is in line with recent guidelines of a joint ATS/ERS task force on outcome measurements in COPD that recommend taking a multi-outcome approach [46]. At the same time, comparison with the cost-effectiveness of other interventions for other diseases is made possible through the calculation of costs per QALY. Uncertainty around cost-effectiveness ratios will be dealt with in probabilistic sensitivity analysis in which costs and health outcomes will be bootstrapped and plotted on cost-effectiveness planes from which cost-effectiveness acceptability curves will be drawn [47-49]. In additional ‘net monetary benefits’ [50] will be calculated using different thresholds of the willingness to pay for a QALY and it will be investigated which patient, practice and team characteristics are related to the size of the net monetary benefits. The economic evaluation will compare differences in costs to differences in effects (CEA) and quality adjusted life-years (CUA). The analysis will have a 12 and 24-months time horizon. Sensitivity analyses will be performed on the perspective (societal versus healthcare) and the applied utility measure (Dutch EQ5D).

## Baseline results

### Primary care practices

The characteristics of the enrolled 54 general practices, which formed 40 clusters, are shown in Table 3. Numbers of included patients per participating cluster ranged from 11 to 79 patients. Most practices were single-handed (44%) or one or more partner practices (41%). The enrolled practices included a total of 76 participating GPs; the majority (61%) were males with a mean age of 50 (range 35–62) years and 16 (SD 8.2) years of practicing.

**Table 3:** Characteristics of included primary care practices in the RECODE study

**General practices**	
Number of GP practices	54
Number of clusters	40
Number of included patients per participating cluster, range	11-79
Type of practice,%	
Single-handed practice	44
One or more partner practice	41
Healthcare centre	15
Practice location,% urban	72
Patient practice population, n (range)	3418 (1750-16907)
Ethnic minorities,%	15
**General practitioners**	
Number of participating GP’s	76
Gender GP,% male	61
Age GP, years (range)	50 (35-62)
Years practicing, years (SD)	16 (8.2)

## Current level of care of the practices

We assessed the current level of COPD care at baseline in all general practices to be able to report any difference in quality of care after 12 months. Results at baseline are shown in Table 4. Almost half of the RECODE patients (53%) have a registered smoking status; however, a standard spirometry test in the last year was less common, with only (12%) of the patients receiving spirometry.

**Table 4:** Description of current level of care of included GP practices: distribution of the performance indicators of the practices

**Measurement category**	**Process indicator**	**% (SD)**
Smoking	% RECODE patients with registered smoking status	53 (27.9)
	% RECODE patients that are registered smokers	35 (19.3)
	%RECODE patients, which are registered smokers with stop-smoking advice in the last year	35 (34.3)
BMI	% RECODE patients of which the BMI is measured in the last year	42 (23.8)
Treatment & monitoring	% RECODE patients with inhalation technique controlled in the last year	13 (20.3)
	% RECODE patients with a spirometry test in the last year	12 (14.9)
	% RECODE patients with monitored functioning with a structured method ( CCQ or MRC) in the last year	28 (27.4)
	% RECODE patients with controlled physical activity in the last year	30 (24.9)

### Patient recruitment

**Figure 2:**
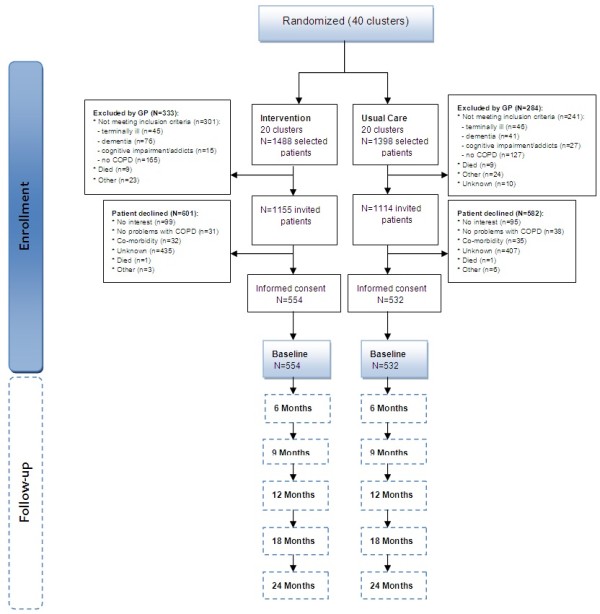
Flowchart of the recruitment to the baseline assessment of the RECODE study.

Figure 2 shows the study flow chart until baseline. In total, 2886 patients were selected in 40 clusters of which 617 (21%) patients were excluded by their GP. Most of these excluded patients were registered as a COPD patient in the EMR; however, after evaluation they turned out to be mislabelled by their GP. After exclusion, 2269 patients were invited to participate, of which 48% participated (response 48%). Most patients indicated no reason for refusing (71%), while others expressed no interest (16%), did not consider themselves to be a COPD patient (6%), or reported not having troublesome COPD symptoms (6%). In total, we have been able to allocate 1086 COPD patients at baseline: 554 participants to the intervention group and 532 participants to the control group. Patients were included from September 2010 until September 2011.

### Non-participation analysis

As we invited all eligible participants for this trial with an invitation letter with an attached CCQ questionnaire, we were able to determine any differences between participants of the trial and COPD patients eligible but declining randomization, in order to assess external validity (Table 5). Of all eligible patients who were invited to participate, 1549 questionnaires had analyzable data. We received a higher response rate (961 vs. 588) of returned CCQ questionnaires in the group of patients willing to participate in the trial, compared to patients eligible but declining randomization. There was no difference in age between both groups. Significantly more men (54.7%) are participating in the RECODE trial compared to the proportion of men in patients who declined participation (46.9%). Furthermore, participants in the trial reported significantly more symptoms and disabilities on their functional and mental state, which was reflected in a mean total CCQ score of 1.8 (1.1), compared to 1.5 (1.1) in non-participants.

**Table 5:** Characteristics and comparison of participants and non-participants of the RECODE trial

	Participant (n = 961)*	**Non-participant (n = 588)**	**p-value**
**Age, years (SD)**	68.7 (11.0)	67.8 (11.5)	0.162
**Males,%**	54.7	46.9	0.003
**CCQ**			
Symptoms	2.4 (1.2)	1.9 (1.2)	<0.001
Functional state	1.8 (1.3)	1.5 (1.4)	<0.001
Mental state	0.9 (1.2)	0.7 (1.2)	<0.001
**Total score**	**1.9 (1.1)**	**1.5 (1.1)**	<0.001

*Values are means (S.D.) unless stated otherwise. ** Of the 1086 RECODE patients, there were 961 CCQ questionnaires available at the time of initial invitation.

### Baseline characteristics COPD patients

Table 6 presents the baseline demographic and clinical characteristics of the included COPD population. Enrolled subjects were mainly elderly (ex) smokers, and had moderate COPD which is reflected by a mean post-bronchodilator FEV1 of 68% predicted. We included COPD patients with substantial co-morbidities: 36.8% had a diagnosis of hypertension, 16.1% suffered from major cardiovascular disease, 14.7% had diabetes and 9.9% had a combined diagnosis of depression. Mean SGRQ total score was 35.6 (20.5) and mean CCQ total score was 1.5 (0.97). The proportion of patients with dyspnea on moderate exertion or worse (MRC score >2) comprised one third of the study population.

**Table 6:** Baseline demographic and clinical characteristics of the patients with COPD included in the RECODE study

	**Total (n = 1086)**
**Men,%**	53.9
**Age, y**	68.3 (11.2)
**Employment,%**	28.3
**Low education,%**	40.3
**Pulmonary function**^**1**^	
Predicted FEV1**%	67.8
FER***%	57.7
GOLD-stage,%^****^	
I Mild	24.6
II Moderate	53.2
III Severe	19.4
IV Very severe	2.9
**Smoking status,%**	
Current	36.7
Former	53.2
Never	10.1
**Co-morbidities**	
Major cardiovascular disease,%	16.1
Hypertension,%	36.8
Diabetes,%	14.7
Depression,%	9.9
Charlson co-morbidity index	2.3 (1.3)
**CCQ**	
Symptoms	2.09 (1.21)
Functional state	1.40 (1.22)
Mental state	0.51 (0.98)
Total score	1.50 (0.97)
**MRC**	
score ≤2.%	66.6
score >2.%	33.4
MRC score (mean)	2.01 (1.28)
**SGRQ**	
Symptom	50.5 (20.9)
Activity	47.8 (29.5)
Impact	23.3 (19.6)
Total	35.6 (20.5)
**EQ-5D**	
Total score	0.74 (0.26)
EQ-VAS	67.0 (17.4)
**SF-36**	
Physical	38.3 (10.8)
Mental	48.6 (10.4)
**IPAQ**	
Total MET minutes	2925 (4683)
High physical activity,%	11.1
Moderate physical activity,%	0.6
Low physical activity,%	88.4
**Self-management**	
Taking initiatives	57.0 (17.9)
Investment behavior	60.4 (17.6)
Self-efficacy	65.3 (17.4)

^*^Values are means and corresponding standard deviations (SD) unless stated otherwise. **FEV1 predicted: Forced expiratory volume in 1 second, post-bronchodilator, predicted according to age and height. ***FER: forced expiratory ratio (FEV1 / FVC x 100%), FVC: forced vital capacity. ^****^Mild = FEV1 > 80%, Moderate = 50% ≤ FEV1 < 80%, Severe = 30% ≤ FEV1 <50%, Very severe = FEV1 < 30%

1. Lungfunction was missing in 66 patients (34 control patients; 32 intervention patients).

## Discussion

Optimal COPD management continues to be an important area of research, as the worldwide prevalence is growing and costs will rise in coming decades. Furthermore, in contrast to asthma patients, medication has demonstrated to have limited effect in the management of COPD patients. IDM for chronic diseases has the potential to influence health status, while reducing total costs [6]. However, the (cost) effectiveness of IDM in primary care COPD patients remains unknown, due to a paucity of randomized clinical trials in this field. This article presents the design and baseline results of the RECODE trial, which aims to assess the (cost) effectiveness of IDM for COPD patients in primary care.

We have chosen a cluster-randomized design to prevent cross-contamination of the IDM intervention within a practice. In order to enhance comparability between the intervention and control group at baseline, clusters were matched by stratification and randomized by a blinded researcher. We were able to allocate a broad sample of 1086 COPD patients (ranging from mild to very severe patients) with a response rate of participants of almost 50%. We can conclude from our non-participation analysis that we have recruited a sufficient proportion of patients with considerable complaints, and thus room for improvement. Furthermore, the included practices showed great diversity in the kind of practice, practice size and distribution of ethnic minorities, thereby contributing to high external validity.

To date, previous clinical trials of disease management or home-based rehabilitation trials in primary care have revealed encouraging results on quality of life [51-55]. Based on an earlier example of a published protocol [56], we compared several aspects of our current study to the previously conducted randomized trials which aimed to evaluate the effectiveness of such programs in primary care or in the home-based setting (Table 7).

**Table 7:** Characteristics of trials evaluating IDM programmes in primary care or home-based setting

	**RECODE**	**Rea 2004**	**Boxall 2005**	**Fernandez 2009**	**Wetering 2010**	**Gottlieb 2011**
**Recruitment**	P	P + S	P + S	S	S	P
**Pilot study**	+	-	-	-	-	-
**Population**	GOLD stage 1-4	GOLD stage 1-4	GOLD 4	GOLD 4	GOLD 2-3	GOLD 2
**Intervention**	Multidisciplinary team training, designing practice and patient relevant treatment plans including education, smoking cessation, physiotherapeutic reactivation, dietary intervention (24 mo)	Exacerbation action plan, structured follow-up by nurse, GP. Education about smoking cessation, medication (12 mo)	Home rehabilitation programma (12 wks), under supervision of physiotherapist. Educational sessions for patients and carers, including structured follow up by physiotherapists, nurses, occupational therapy	Home-rehabilitation programme (11 mo) under supervision of physiotherapist. Three education sessions	Intensive exercise programme (4mo), individualized education programme, smoking cessation, dietary intervention (if needed). 20mo maintenance phase, exercise at home (under supervision).	Intensive exercise and educational programme (7wks) led by multidisciplinary team. Smoking cessation counseling.
**Included HCP**	3-5	3	3	2	3	?
**Randomization**	Clustered	Clustered	Individual	Individual	Individual	Individual
**Blinding outcome** **assessor**	+	-	-	-	+	-
**Stratification/matching**	+	-	-	-	-	-
**Powercalculation based on**	MRC score >2	Hospital days	6MWD	Not mentioned	SGRQ	Not mentioned
**Cost-effectiveness analysis**	+	-	-	-	+	-
**Included patients**	1086	135	60	50	199	61
**Follow-up (months)**	24	12	3	12	24	18

### Selection of patients

In respiratory medicine there is a lack of research on mild to moderate COPD patients, despite that over 80% of COPD patients suffer from this stage of disease and are often treated in primary care. Moreover, it has been shown that treatment decisions for asthma and COPD patients are usually based on studies including a very small and highly selected proportion of the real patient population; this indicates the need for more real-life studies targeted at the true population, and applying less exclusion criteria [57]. Former trials included a highly selected severely ill patient population [51,52] or recruited their patients in secondary care [55]; overall, this is not an uncommon phenomenon in primary care COPD trials.

### Limited follow-up

Most studies presented data up to 12 months follow-up, while limited information is available on studies with long-term (18 or 24 months) follow-up. Gottlieb et al. evaluated the effect of an intensive exercise and educational program in patients with moderate COPD during 18 months of follow-up [53]. Although an effect was found on walking distance and quality of life, the effect on quality of life disappeared over 18 months. However, this result should be interpreted with caution, as the intensive rehabilitation program lasted only 7 weeks, which was followed by a maintenance phase including a monthly session focusing on ways of incorporating exercise in daily life. Furthermore, the authors acknowledged many dropouts before randomization, at randomization and during rehabilitation, potentially introducing bias and indicating substantial loss of power [53]. Another study evaluated the efficacy of a community-based COPD management program in less advanced (GOLD 2 and 3) COPD patients during 24 months follow-up. The SGRQ score initially improved in the intervention group compared to the control group. At 12 months, scores in the intervention group had returned to baseline, whereas in the usual care group it remained stable up to 12 months and worsened thereafter [55].

### Methodological aspects

Due to the nature of the intervention, blinding of participants and patients to the intervention is usually impossible. However, blinding of an outcome assessor can substantially diminish the risk of bias. All the above-mentioned studies, except for the trial of Wetering et al. [55], failed to introduce blinded outcome assessors or did not report this as such. In the study of Rea et al. [54], randomization was also clustered, comparable to our study; however, statistical analysis was at the level of the patient, thereby not taking the clustering coefficient in account. Furthermore, the authors failed to allocate five practices to the correct treatment group.

### Planned subgroups

Finally, this study differs from the other studies in that we based our sample size estimates on the a priori planned subgroup of patients with an MRC dyspnea score >2. We earlier reported that we found the greatest improvements on quality of life in these patients [25]. It is probably that lung function is still relatively well maintained at this stage, while patients experience considerable dyspnea and an impaired quality of life [20]. As a result of this pre-planned subgroup power analysis and to compensate for the intra-clustering, we allocated almost 1100 patients in the present trial according to protocol. As can be seen in Table 7, this number is much higher than that of earlier studies in this field.

## Conclusion

It is acknowledged that not all patients who potentially benefit from an exercise training program, pulmonary rehabilitation, or smoking cessation intervention are actually receiving this type of support in daily practice. It is likely that costs will be lower when patients are detected and persuaded to change their lifestyle at an earlier stage, possibly reducing health decline and disease progression in the long term. To the best of our knowledge, this is the first and largest cluster randomized trial to evaluate the cost and clinical effectiveness of IDM in primary care COPD patients. The results of this study will provide insight into the clinical and cost-effectiveness of IDM in primary care COPD patients, also on the long term.

## Abbreviations

ACIC: Assessment Chronic Illness Care; BMI: Body Mass Index; CCQ: Clinical COPD Questionnaire; CCI: Charlson Comorbidity Index; CEA: Cost Effectiveness Analysis; COPD: Chronic Obstructive Pulmonary Disease; CUA: Cost Utility Analysis; EMRs: Electronic Medical Records; EQ-5D: EuroQol-5D; FEV1: Forced Expiratory Volume in one second; FVC: Forced Vital Capacity; GOLD: Global Initiative for Chronic Obstructive Lung Disease; HCP: HealthcCare Providers; ICC: Intra Cluster Coefficient; IDM: Integrated Disease Management; IPAQ: International Physical Activity Questionnaire; GP: General Practitioner; LEON: Leiden Primary Care Research Network; MCID: Minimum Clinical Important Difference; MET: Metabolic Equivalent Time; MRC: Medical Research Counsil; PACIC: Patient Assessment Chronic Illness Care; PR: Pulmonary Rehabilitation; QALY: Quality Adjusted Life Years; RCT: Randomised Controlled Trial; SF-36: ShortForm-36; SGRQ: Saint Georges Respiratory Questionnaire; SMAS-30: Self Management Scale-30.

## Competing interests

The authors declare that they have no competing interests.

## Authors’ contributions

All authors are part of the RECODE research team. MR and NC are the principal investigators, supervisors and grant applicators of the cost and clinical effectiveness part of this study, respectively. AK is investigator of the clinical part of the study and wrote the final manuscript, IS assisted and wrote the first draft of the manuscript as part of a student project. AT and MB are investigators of the cost-effectiveness part of the study and wrote the economic and cost-effectiveness parts of the manuscript. AK, IS and MB collected the data and performed data analyses, with assistance of AT. JG and WA are supervisors and advised in the design of the study and the preparation of the manuscript. All authors read, edited and approved the final version of the manuscript.

## Pre-publication history

The pre-publication history for this paper can be accessed here:

http://www.biomedcentral.com/1471-2466/13/17/prepub
